# Lethal thrombosis of the iliac artery caused by *Aspergillus fumigatus* after liver transplantation: case report and review of the literature

**DOI:** 10.1186/s12893-019-0668-4

**Published:** 2019-12-27

**Authors:** Jan-Paul Gundlach, Rainer Günther, Helmut Fickenscher, Marcus Both, Christoph Röcken, Thomas Becker, Felix Braun

**Affiliations:** 1 Department of General, Visceral-, Thoracic-, Transplantation- and Pediatric Surgery, University Medical Center Schleswig-Holstein (UKSH), Campus Kiel, and Christian-Albrecht University (CAU), Arnold-Heller-Str. 3, 24105 Kiel, Germany; 2 Department of Internal Medicine I, UKSH and CAU, Campus Kiel, Kiel, Germany; 3 Department of Infection Medicine, UKSH and CAU, Campus Kiel, Kiel, Germany; 4 Department of Radiology and Neuroradiology, UKSH and CAU, Campus Kiel, Kiel, Germany; 5 Department of Pathology, UKSH and CAU, Campus Kiel, Kiel, Germany

**Keywords:** *Aspergillus fumigatus*, Liver transplantation, Arterial thrombosis

## Abstract

**Background:**

*Aspergillus fumigatus* infections frequently occur after solid organ transplantation. Yet, a fungal thrombosis after liver transplantation is an exceptional finding.

**Case presentation:**

We report on a 44-year-old female with an aspergillosis after liver transplantation for autoimmune hepatitis. On postoperative day (pod) 7, seizures occurred and imaging diagnostics revealed an intracranial lesion. Anidulafungin was initiated in suspicion of mycosis and switched to voriconazole on suspicion of an Aspergillus spp. infection. Progression of the cerebral lesion prompted craniotomy (pod 48) and the aspergillosis was verified. The patient was discharged with oral voriconazole therapy. Re-admission was necessary with acute-on-chronic renal failure after a tacrolimus overdose on pod 130. The patient received a pelvic angiography due to a temperature difference in the legs. It showed a complete iliac artery thrombosis which was subsecutively surgically removed. The histopathological examination revealed an *Aspergillus fumigatus* conglomerate. The patient died on pod 210 due to systemic aspergillosis.

**Conclusion:**

The acute development of focal neurologic deficits is common in patients with an aspergillosis of the brain. Nevertheless, arterial thrombosis after *Aspergillus fumigatus* is less frequent and, to the best of our knowledge, its occurrence after liver transplantation has not yet been reported so far. Due to its rarity, we added a review of the literature to this manuscript.

## Background

Fungal infections frequently occur after solid organ transplantation (SOT). Aspergillus (A.) species account for a significant proportion of these infections, especially *A. fumigatus*, which is a contaminant of the respiratory tract. *A. fumigatus* produces airborne conidia that can cause infections in immunosuppressed patients [1]. Infections can involve the lungs, paranasal sinuses, and orbital bones, appearing as chronic granulomatous disease or disseminated infection characterized by tissue necrosis. Invasive aspergillosis is characterized by vascular invasion and thrombosis. Central nervous system (CNS) involvement regularly occurs through hematogenous spread from pulmonary infections. At the same time, CNS involvement mostly affects immunocompromised patients, is often accompanied by generalized infections [2] and usually correlates with poor prognosis [3, 4].

The outcome of infections due to aspergillus species in SOT recipients has been poor. After liver transplantations (LT), cases of invasive aspergillosis have been reported with disastrous outcome: the overall 1-year cumulative survival probability was reported as 35% [4]; and long-term survival remains rare [3, 5]. The majority of *A. fumigatus* infections occur in the first 6 months after transplantation, when immunosuppression is at its highest level [4].

*A. fumigatus* infections are difficult to diagnose due to the low rate of positive cultures. The galactomannan antigen test from serum samples may allow an early diagnosis even if cultures remain negative due to antifungal treatment. The rarity of this pathology makes its therapeutic treatment very difficult on a multidisciplinary level. The acute development of focal neurologic deficit is common in patients with aspergillosis of the CNS and cerebral imaging diagnostics often reveals fungal inflammation [6]. In our described case, the diagnosis of cerebral aspergillosis was supposed after the development of focal seizures and was verified histologically after surgical removal. Moreover, arterial thrombosis after *A. fumigatus* is less frequent and to the best of our knowledge, its occurrence after LT has not yet been reported so far.

## Case presentation

A 44 years old female Caucasian was diagnosed with autoimmune hepatitis. Histologically, giant cell hepatitis was discussed. The disease did not respond to a high dosage of steroids (100 mg for 5 days with consecutive reduction) with acute-on-chronic decompensation and the patient was evaluated and listed for LT. The Model of End Stage Liver Disease score (Lab-MELD) was 33 and the Child-Pugh score was C. Liver transplantation was performed 5 weeks after first admission in January 2015 with a full-size graft of 1200 g. The female donor was 72 years old. The cytomegalovirus status was D+/R- and the cold ischemia time lasted 10 h 8 min. The donor received 11 red blood cell concentrates, five platelet concentrates, and 18 fresh frozen plasma doses. At the time of LT, microbiological smears showed negative results. Reoperations with lavage were needed after initial liver packing. Already on pod 6, cultures from tracheal and bronchial samples yielded growth of Aspergillus spp., and Aspergillus galactomannan antigenemia was demonstrated from pod 0 to 39. During the concomitant postoperative time, the patient suffered from an aspergillus infection which was primarily suspected after cranial imaging and subsequently detected following the craniotomy due to the abscess removal.

**Fig. 1:**
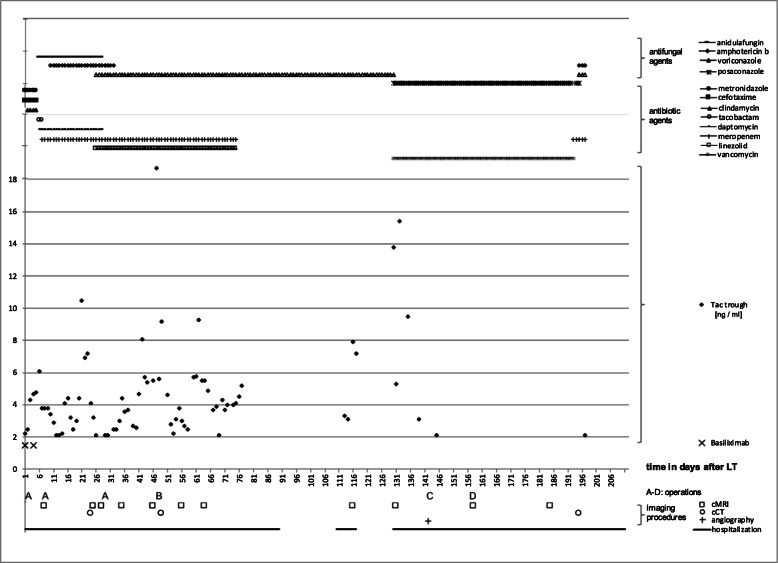
Postoperative tacrolimus trough. Antimicrobial treatment, operations, imaging procedures and hospitalization are additionally shown. *A*: additional laparotomies i.e. removal of abdominal cloths, abdominal lavage, and closure of burst abdomen, respectively. *B*: craniotomy. *C*: thrombectomy. *D*: vitrectomy

Initial immunosuppression consisted of a steroid induction dosage of 500 mg prednisolone administered perioperatively, a basiliximab induction 20 mg i.v. on pod 0 and 4, followed by a low dose tacrolimus and a prednisolone taper schedule (starting dosage of 20 mg). The tacrolimus dosage was achieved to a trough level in the range of 4–6 ng/ml (Fig. 1).

The standard antibiotic treatment was escalated based on the evidence of pneumonia within computed tomography (CT) diagnostics with piperacillin, tazobactam and meropenem, and was switched to daptomycin after vancomycin-resistant enterococci (VRE) had been isolated repeatedly (Fig. 1). Antifungal therapy was not applied as a standard treatment. An intermittent haemodialysis was started on pod 5 due to acute renal failure after LT and continued for 3 weeks, until spontaneous diuresis returned.

**Fig. 2:**
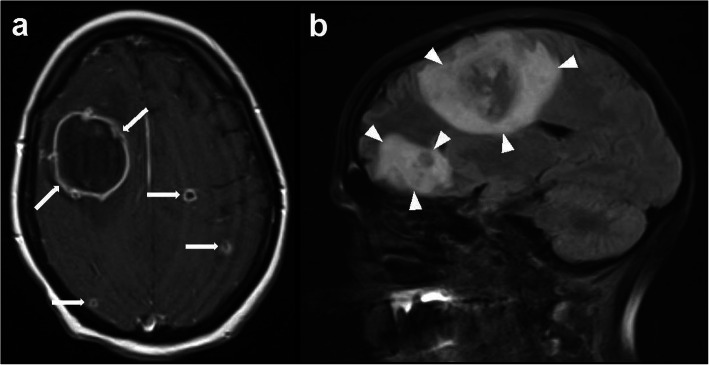
Cerebral MRI reveals multiple mycotic lesions with peripheral contrast media uptake (**a**: transverse T1 weighted spin echo sequence, *arrows*) and surrounding edema (**b**: sagittal FLAIR sequence, *arrowheads*) before craniotomy and subsequent abscess removal

Focal seizures occurred on pod 9. Subsequent cranial diagnostics demonstrated an expansion of the lesion to the frontal lobe. Further differentiation in cranial magnetic-resonance imaging (cMRI) revealed multiple cerebral lesions (Fig. 2). A calculated therapy was started with anidulafungin on suspicion of an invasive fungal infection. Repeated cMRI scans were performed (Fig. 1). Following, alongside increasing infectious parameters, progressive unilateral symptoms and somnolence developed. Nevertheless, repeated MRI showed unaltered findings. The symptoms were classified as septic encephalopathy. The antimicrobial therapy was escalated again, and the aforementioned antifungal therapy was changed to voriconazole. Immunosuppression was administered with a continuous hydrocortisone infusion.

With further neurologic exacerbation, the MRI showed progressive cerebral disease. Hence, craniotomy and abscess removal were executed on pod 48. Microbiological analyses confirmed invasive fungal infection with *A. fumigatus*. Hereupon, neurologic symptoms slowly improved. Three months posttransplant, the patient was discharged from hospital. The antimicrobial prophylaxis was cotrimoxazole, valganciclovir and amphotericin B, as well as a continued antifungal therapy with voriconazole.

In the time that followed, the patient returned to hospital for 1 week because of gastroenteritis and cytomegalovirus DNA load (DNemia from pod 74 to 117). Four months posttransplant, the patient was hospitalized again due to acute-on-chronic renal failure with exsiccosis and hyperkalemia after a tacrolimus overdose. With widespread intensive care management, dialysis was evaded. The cMRI suggested intracerebral abscess progress, thus, the antifungal therapy was switched to posaconazole.

**Fig. 3:**
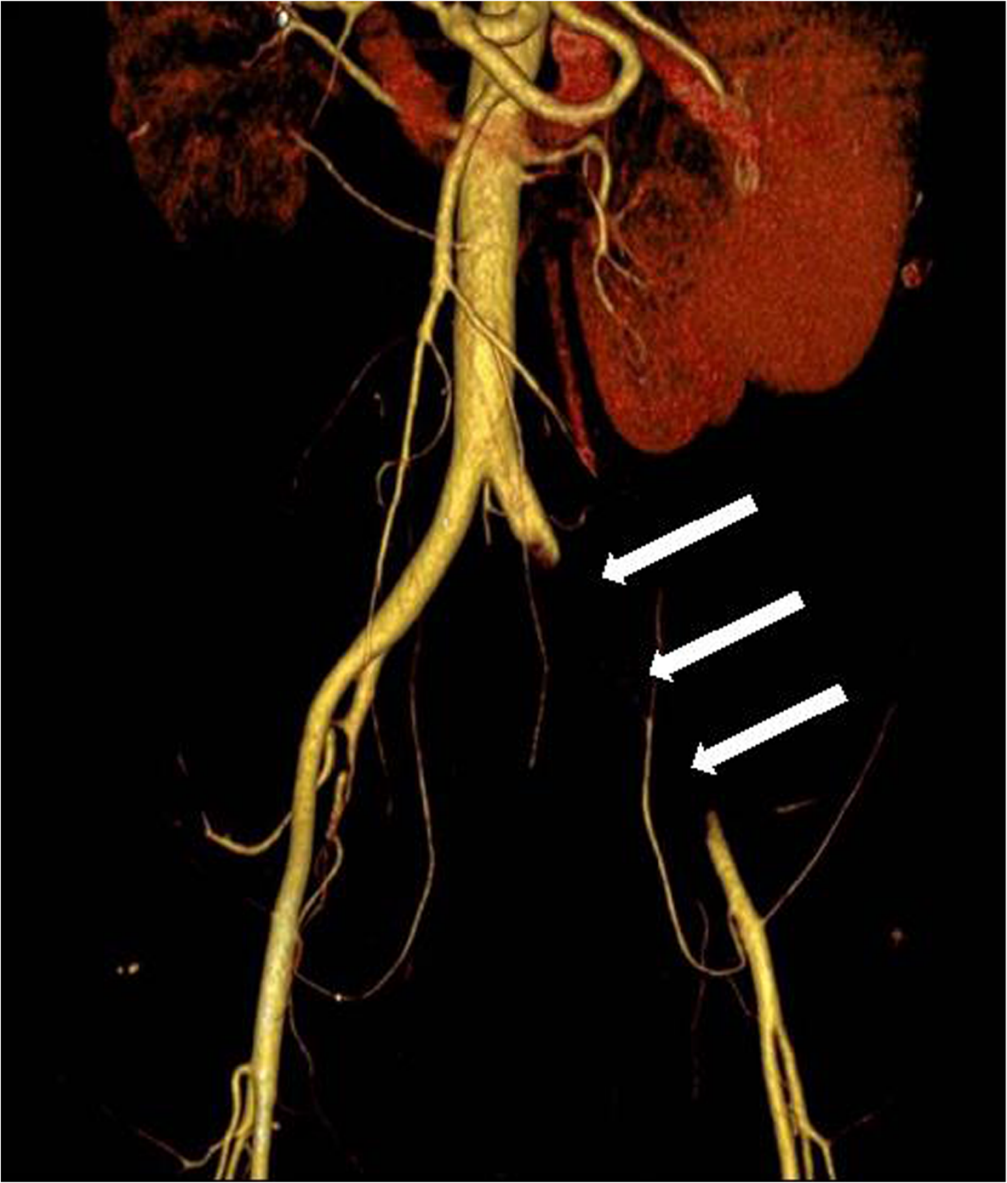
Occlusion of the left common and internal iliac artery (*arrows*) is detected by CT-angiography

**Fig. 4:**
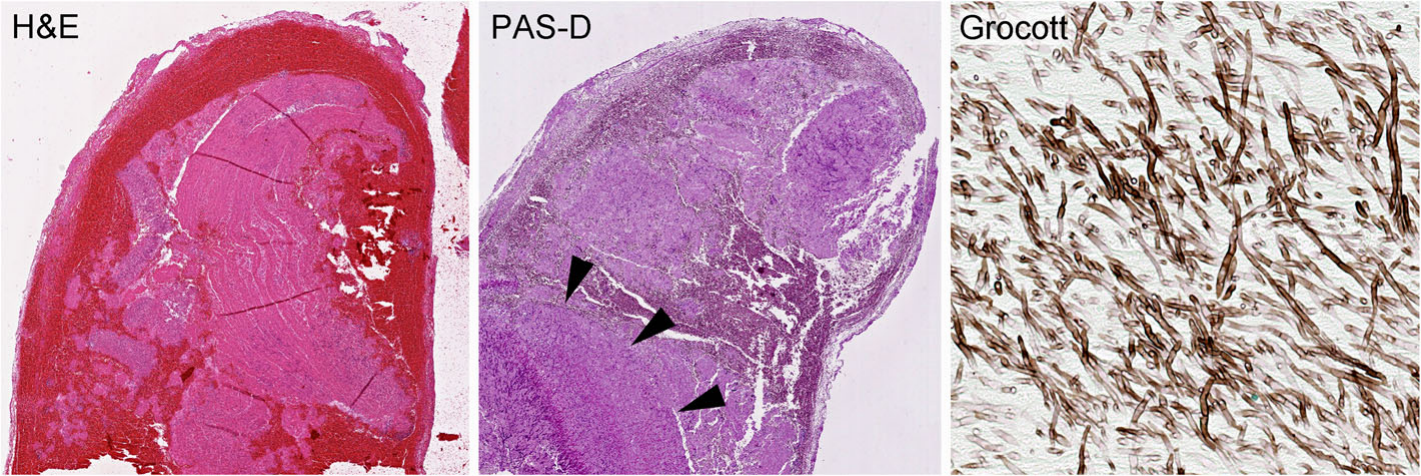
Histomorphological analysis of conglomerate after thrombectomy. Hematoxylin and eosin (H&E) stained section, as well as PAS staining showed mycotic inflammation, respectively (both × 40). Fungus demonstration by Grocott staining (× 400). Note: *Arrowheads* were used to indicate relevant areas of mycelium. Subsequent DNA analysis verified *A. fumigatus*

Five months posttransplant (pod 142), a temperature difference in the legs were noticed. In the following CT angiography (Fig. 3), an occlusion of the left common iliac artery (CIA) and of the left internal iliac artery was detected. After the subsequent operative thrombectomy on the following day, histopathology proved the presence of *A. fumigatus* in the thrombus (Fig. 4). Additionally, after notice of visual impairment, an invasive fungal infection of the vitreous body was diagnosed and a vitrectomy was executed. The visual capacity improved satisfactorily afterwards.

Under hydrocortisone, the serum bilirubin levels increased and rejection was histologically diagnosed (RAI 6) on pod 195. Galactomannan antigenemia was demonstrated again, starting on pod 197. The antifungal therapy was changed to voriconazole and amphotericin B. A variceal hemorrhage occurred and the patient was transferred to our intensive care unit. After all therapeutic approaches had been exhausted, the patient was moved to our palliative care ward and died 7 months (pod 210) posttransplant.

## Discussion and conclusions

Although reports on *A. fumigatus* associated thrombosis following kidney transplantation have been reported [7], fungal thrombosis following orthotopic liver transplantation (LT) is unique to the best of our knowledge. We found one case of portosplenomesenteric thrombosis following LT after pneumonic superinfection with *A. fumigatus*, but, without evidence for fungal thrombosis [8]. In addition, intracerebral thrombotic complications following *A. fumigatus* infections in pediatric LT have been reported [9].

### Immune deficiency supports *Aspergillus fumigatus* infections and thrombosis

Arterial thrombosis due to *A. fumigatus* infection is an exceptional complication of invasive aspergillosis in immunocompromised patients. Aspergillus is a ubiquitous fungus in the air [1] and consequently patient’s infection usually occurs via the respiratory system as a silent carrier leading to pneumonia [10]. In our case, no other events of systemic aspergillosis were recorded in the hospital at that time and the patient was healthy prior to LT. Subsequently, an invasive fungal spread from the patient’s respiratory tract through the bloodstream is considered to be the likely source of infection. Apart from postoperative intensive care medicine and immunosuppression, no other known risk factors for invasive aspergillosis - such as chronic pulmonary diseases - were present [11]. In detail, several risk factors for thrombosis due to *A. fumigatus* infections have been identified: The major predisposing factors include prolonged neutropenia, chronic administration of corticosteroids [12, 13], as well as the insertion of prosthetic devices [14, 15] and tissue damage due to prior infection or trauma [10]. In immunocompromised patients, the immune system fails to eliminate *A. fumigatus’* conidia which results in hyphae multiplication and dissemination [16].

### Antifungal answer: platelets cure but also clog

The human antifungal protection system consists of different cellular compounds, making itboth a blessing and a curse: platelets participate by inhibiting the invasion-supporting germination and hyphal elongation [17], and become activated [18]. The binding to hyphae is an important step in the immune defense against aspergillus since platelets store antimicrobial peptides and serotonin which have antifungal effects themselves. Nevertheless, platelets are unable to internalize conidia but adhere and cover the fungus [17]. Hence, the mutual impact with human platelets results in thrombosis and consequently tissue infarction.

### Limited diagnostic prospects and recent treatment options

Establishing the diagnosis is difficult due to the lack of positive blood cultures in most cases, nonspecific clinical presentation, varying clinical course and lack of definitive imaging characteristics. Furthermore, no optimal strategy exists concerning the timing of surgically intervention or the duration of conservative treatment. The serum galactomannan antigen test allowed the early detection of aspergillosis; however, false-positive results may hinder its benefit [4]. Elevated galactomannan antigen levels were detectable again only in the very terminal phase.

From 2005, some antifungal agents such as itraconazole and deoxycholate amphotericin B were no longer considered first-choice drugs for invasive aspergillosis: Therapy is hindered by CNS aspergillosis in immunocompromised patients, which has shown excellent response to voriconazole so far. The use of voriconazole was significantly associated with a higher probability of survival (*p* < 0.001) in a review including 116 patients suffering from invasive aspergillosis after LT [4]. The overall survival has improved in the last decade since combined therapies were proven to be beneficial [4]: similarly, combination regimens with voriconazole were beneficial for survival (p < 0.001) [4] and complementary effects were shown for a combination of voriconazole and caspofungin being independently associated with reduced mortality in patients with *A. fumigatus* infections [19].

In LT, caspofungin is associated with a significantly higher risk of adverse events, especially acute renal failure (*p* = 0.001), even though it does not carry an apparent increase in the risk of death or acute cellular rejection in LT [20]. Thus, voriconazole is the preferred antifungal therapy in SOT, regardless of possible influence in the tacrolimus metabolism in human liver microsomes [21]. Nevertheless, drug interactions of antifungal therapy and immunosuppression must always be considered. Our switch of antifungal therapy to posaconazole should be critically reviewed. For breakthrough infections, liposomal amphotericin B or echinocandin should have been preferred [22]. Furthermore, echinocandins are proposed for prophylaxis of invasive fungal infections in high-risk patients because of their coverage of Candida, Aspergillus and *Pneumocystis* pneumonia, their low nephrotoxicity and hepatotoxicity, rare drug–drug interactions and low risk of drug resistance [23]. Future investigations are needed to determine the role of combined approaches for invasive aspergillosis in LT.

In conclusion, cerebral aspergillosis accompanied by invasive fungal arterial thrombosis represents a critical problem for the liver transplant recipient, with a highly lethal course. Its diagnosis and therapy need to be quick and precise.

## Data Availability

The clinical datasets supporting the conclusions of this study were derived from the patient files (paper and electronic form). Therefore, restrictions to availability apply due to data protection regulations. Anonymized data are, however, available from the corresponding author on reasonable request and with permission of the University Hospital Schleswig-Holstein and the local review board.

## Abbreviations

cMRI: cranial magnetic-resonance imaging
CNS: central nervous system
LT: liver transplantation
pod: postoperative day
MELD: Model of End Stage Liver Disease score
RAI: rejection activity index
SOT: solid organ transplantation
